# Natural History of Clinical Phenotypes and Their Biochemical Correlates in Adult X‐Linked Adrenoleukodystrophy

**DOI:** 10.1002/jimd.70176

**Published:** 2026-03-19

**Authors:** Julia Lier, Markus Ponleitner, Denny Popp, Lisa Schäfer, Franziska Küstermann, Christof Meigen, Frank Kratzer, Joachim Janda, Jürgen Okun, Rami Abou Jamra, Wolfgang Köhler, Caroline Bergner

**Affiliations:** ^1^ Department of Neurology University Medical Center Leipzig Germany; ^2^ Department of Neurology Medical University of Vienna; Comprehensive Center for Clinical Neurosciences and Mental Health Vienna Austria; ^3^ Institute of Human Genetics, University of Leipzig Medical Center Leipzig Saxony Germany; ^4^ LIFE Leipzig Research Center for Civilization Diseases, Leipzig University Leipzig Germany; ^5^ German Center for Child and Adolescent Health (DZKJ), Partner Site Leipzig/Dresden Leipzig Germany; ^6^ Division of Neuropediatrics and Metabolic Medicine, Department of Pediatrics I Center for Pediatric and Adolescent Medicine, University Hospital Heidelberg Heidelberg Germany

**Keywords:** data registry, leukodystrophy, natural history, x‐ALD

## Abstract

X‐linked adrenoleukodystrophy (X‐ALD) is a rare monogenic disorder characterized by marked variability in clinical presentation, age at onset, and disease progression. A deeper understanding of its natural history and the relationship between biochemical markers and clinical phenotypes is essential for improving disease monitoring, patient counseling, and optimizing clinical trial design. In particular, the predictive value of very long‐chain fatty acids (VLCFA) for clinical phenotypes has recently garnered increased attention. In this longitudinal, mixed prospective/retrospective, single‐center study, we analyzed clinical and biochemical data from 364 patients with X‐ALD (255 males). Parameters included clinical scores (EDSS, AACS), age at symptom onset, and disease manifestations, which were correlated with individual VLCFA levels. Patients with adrenal insufficiency (AI) exhibited significantly elevated VLCFA concentrations. Higher C26:0 levels were associated with faster progression (measured by EDSS); however, effect sizes were small and inter‐individual variability considerable. Although initial symptom severity was comparable between sexes, males presented earlier and progressed faster. Among patients seen in early clinical stages (EDSS ≤ 4.5), disease progression rates were higher (males: 0.34 ± 0.77; females: 0.11 ± 0.11) than in those presenting at more advanced stages (EDSS > 4.5; males: 0.23 ± 0.33; females: 0.09 ± 0.10). We provide comprehensive data on the prevalence of disease manifestations and the natural course of X‐ALD in a large adult cohort. The observed association between elevated VLCFA levels and adrenal insufficiency should be considered in future clinical monitoring and trial design. However, due to the small effect sizes and variability, VLCFA levels offer limited prognostic utility for individual patients.

## 1 Introduction

X‐linked adrenoleukodystrophy (X‐ALD) is a rare monogenic disorder caused by pathogenic variants in the *ABCD1* gene [1]. These mutations disrupt peroxisomal import, leading to impaired beta‐oxidation and subsequent accumulation of very long‐chain fatty acids (VLCFAs) in all body tissues and fluids [2]. X‐ALD presents with distinct clinical manifestations. Adrenomyeloneuropathy (AMN) is characterized by length‐dependent degeneration of spinal cord tracts and peripheral polyneuropathy [3]. All adult men with X‐ALD develop AMN symptoms during their lifetime but the onset of disease may largely differ between individuals [4]. While women are also frequently affected, their symptoms are typically milder and have a later onset [5, 6, 7]. In addition, VLCFA accumulation in the adrenal cortex results in adrenal insufficiency (AI) in up to 60%–70% of affected males [8, 9], necessitating lifelong oral adrenal hormone replacement. The cerebral form of X‐ALD (cALD) involves progressive, inflammatory destruction of cerebral white matter [10]. It occurs almost exclusively in males and is marked by rapid neurological decline, often resulting in death within a few years [8, 11]. Genotype–phenotype correlations as well as predictive biomarkers in X‐ALD have historically been missing. Interestingly, a recent analysis by Jaspers et al. [12] reported that elevated plasma VLCFA levels, specifically C26:0‐lysophosphatidylcholine levels, are associated with increased disease activity in both male and female patients. Multiple studies have demonstrated a cytotoxic role of very long‐chain fatty acids (VLCFAs), showing that they induce mitochondrial dysfunction [13, 14] and increase reactive oxygen species production in patient‐derived fibroblasts as well as in glial cells and oligodendrocytes. Consequently, a mechanistic contribution of elevated VLCFA levels to greater symptom severity appears plausible [15, 16]. The observed association may have important implications, particularly in the context of recent advances in newborn screening, as the ability to predict phenotypes could enable more targeted and refined clinical monitoring [17, 18]. However, significant uncertainty remains regarding the predictive value of VLCFAs at the level of individual patients, and larger cohorts are needed to validate and refine those associations.

Here, we present clinical data from a large, single‐center adult X‐ALD patient cohort. This mixed retrospective/prospective dataset includes 364 X‐ALD patients enrolled in the Leipzig Adult Leukodystrophy Registry with follow‐up periods of up to 25 years, making it one of the largest natural history cohorts published to date. We analyzed plasma VLCFA levels alongside clinical parameters to examine potential associations between biochemical measures and clinical phenotypes. Our comparatively large dataset provides a comprehensive basis for evaluating the utility of VLCFA measurements in guiding clinical decision‐making.

## 2 Methods

### 2.1 Patient Inclusion

The Leipzig Adult X‐ALD cohort is part of the Leipzig Adult Leukodystrophy Registry. In the following, the *prospective cohort* refers to patients that were prospectively enrolled in the registry from 2021 onwards. Retrospective data of these patients was available dating back as far as the year 2000 at maximum. Within the *full cohort*, additional patients were included only retrospectively who were previously treated at the leukodystrophy outpatient clinics in Leipzig and Wermsdorf. To avoid selection bias, as availability of retrospective patient data was influenced by the scope of previous studies, the retrospective cohort only served the analysis of VLCFA levels in specific manifestations, while all other calculations were restricted to the prospective cohort. Diagnosis in men was confirmed through genetic or biochemical testing, whereas carrier status in women was determined either by molecular testing or in obligate gene carriers (female descendants of affected males) inferred from the genotype of the father, consistent with X‐linked inheritance. The study was approved by the local institutional review board at Leipzig University (371/21 ek).

### 2.2 Registry Structure

Storing of identifying data (name, date of birth), pseudonymization of patient data, and record linkage was managed using the in‐house developed software LEIM that relies on solutions similar to those published in Wagner et al. [19]. Clinical data was entered into data capturing elements in the REDCap Data Management tool [20, 21]. Unique datasheets capture demographic data, information about onset of disease and medical history. Longitudinal datasheets are updated at each visit and capture visit‐specific data, including manifestations, dietary or medical treatments, VLCFA levels, and clinical scores.

### 2.3 Neurological Examinations and Disease Manifestations

In the prospective cohort, patients underwent standardized neurological evaluations conducted by neurologists specialized in leukodystrophies at each follow‐up visit. The clinical assessment included a comprehensive neurological examination, documentation of disease manifestation, and the recording of standardized clinical scores, specifically the Expanded Disability Status Scale (EDSS [22]) and the Adult Adrenoleukodystrophy Clinical Score (AACS [23]). The AACS is a semi‐quantitative, disease‐specific composite scale developed to assess the severity and progression of neurological dysfunction in adult patients with X‐ALD. Retrospectively entered clinical scores were based on the review of medical charts, in which EDSS and AACS were obtained during clinical routine. Diagnosis of AI in this study was based on medical history or the presence of lowered cortisol levels in association with clinical signs of AI, leading to oral corticoid replacement. Arrested cALD was defined by the absence of volume growth, as judged by an experienced neurologist/radiologist in visual examinations of consecutive MRI over at least 1 year. Gadolinium enhancement was consistently interpreted as a marker of ongoing disease activity and, therefore, indicative of progressive cALD, even in cases where lesion size remained stable.

### 2.4 VLCFA

VLCFA measurements were collected as part of the standard clinical monitoring and entered in the database. VLCFA levels were analyzed both as patient‐level means and on a per‐visit basis in the full cohort. The VLCFAs were determined in plasma by gas chromatography–mass spectrometry (GC–MS) and their concentrations expressed in μmol/l. Levels in other units (e.g., mg/l) were excluded. All laboratories from which VLCFA results were included in the study successfully participated in suitable interlaboratory proficiency tests, thus assuring the comparability of the results. Reference ranges: C22:0 (21.43–94.69 μmol/L), C24:0 (18.99–72.64 μmol/L), C26:0 (0.00–1.08 μmol/L), C24:0/C22:0 ratio (≤ 1.158), and C26:0/C22:0 ratio (≤ 0.031).

### 2.5 Genotypes

If available, genetic information was recorded for each patient. Information on DNA variants, protein variants, and zygosity was collected in the registry. Genotypes were compared to published variants available in Pubmed, Clinvar, HGMD, and ABCD1 variant registry (https://adrenoleukodystrophy.info/mutations‐and‐variants‐in‐abcd1) and unpublished variants are reported in the Table S2.

## 3 Statistical Analysis

Statistical analysis was performed using R (version 4.4.2, list of packages in Table S1). Normality was assessed with the Shapiro–Wilk test. For comparison of two groups, Mann–Whitney U test or *t*‐test were applied accordingly. For multiple groups, Kruskal‐Wallis test followed by post hoc group comparisons with Dunn's test or ANOVA followed by Tukey's post hoc comparison were used. Multiple comparisons were adjusted with Bonferroni correction, and a *p* value < 0.05 was considered statistically significant. Fisher's exact test was used for group comparisons of categorical variables, and odds ratios calculated accordingly. Correlations were analyzed using Spearman (for non‐parametric data) or Pearson's correlation (for parametric data). Differences between correlation coefficients were evaluated using Fisher's *Z*‐test.

We used linear mixed‐effects models to evaluate the relationship between EDSS scores and biochemical/clinical predictors, accounting for within‐subject repeated measures. The model included fixed effects for time (in years), a predictor (VLCFA plasma levels, manifestation of disease), age at visit, and their interaction (predictor × age at visit). Model diagnostics (residual plots and scaled residuals) were used to assess possible violations of normality. Significance codes: ***p < 0.001, **p < 0.01, *p < 0.05, *p* < 0.10.

## 4 Results

### 4.1 Demographical and Clinical Characteristics of Analyzed Cohort

**FIGURE 1:**
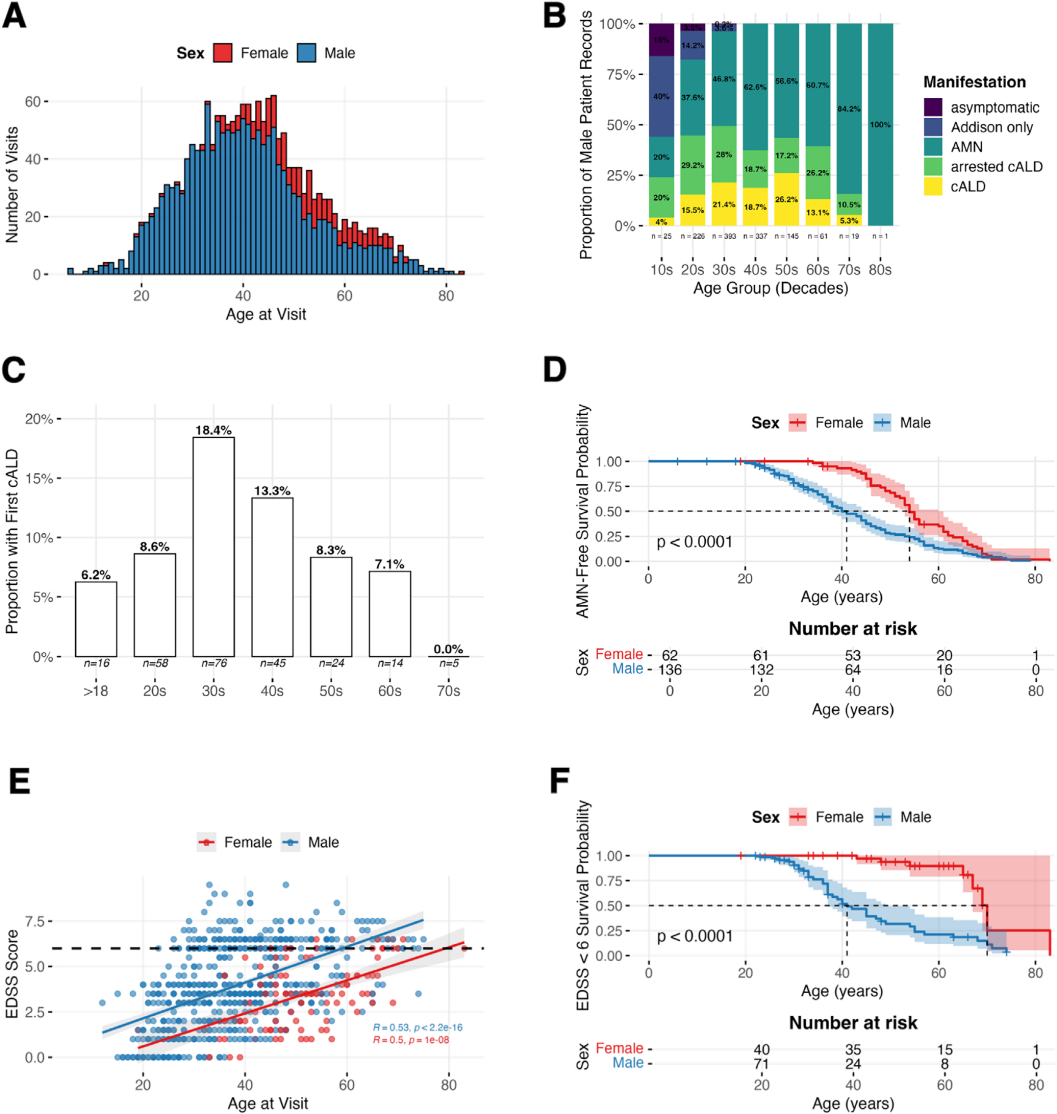
(A) Number of available visits by age stratified by sex. (B) Proportional distribution of manifestations by age decade in male patients. The analysis is visit‐based, the number of visits for each decade is given below the barplot. (C) Proportional distribution of adult cerebral conversion (onset of cerebral disease) by age decade. In our cohort, the majority of adult cerebral conversions occurred between the ages of 30 and 40, with 18.4% of individuals in this age group progressing to cerebral disease (prospective cohort). (D) Kaplan–Meier survival plot showing event‐free survival to AMN manifestation stratified by sex. Censored observations are shown as tick marks. A significant difference between sexes was detected (log‐rank test, *p* < 0.001, prospective cohort). (E) Spearman Correlation of age at visit and EDSS in male and female patients. Male: *ρ* = 0.53, *p* < 0.001; Female: *ρ* = 0.5, *p* < 0.001. Dotted line represents EDSS of 6. (F) Kaplan–Meier survival plot showing survival probability of reaching an EDSS of 6 marking severe gait dysfunction needing assistive devices. Censored observations are shown as tick marks. A significant difference between sexes was detected (log‐rank test, *p* < 0.001, prospective cohort).

A total of 333 visits for female patients and 1764 visits for male patients were analyzed in the study (Figure 1A). Demographic and clinical characteristics and information on follow‐up of the full and prospective cohorts are summarized in Table 1. Within the full cohort, 17.6% of patients received their diagnosis before symptom onset, usually within the context of family screenings. In the remaining patients, the average time until diagnosis was 7.4 ± 7.2 years after symptom onset with no relevant difference between male and female patients (Figure S1A, *p* = 0.43). In our full cohort we detected 84 different genetic variants in ABCD1, out of which 8 have not been previously published and are reported in Table S2.

**TABLE 1:** Demographic and clinical characteristics.

	Male	Female
Full cohort		
*n* patients	255	109
Visits per patient (mean ± SD, min–max)	6.92 ± 5.53, 1–23	3.05 ± 3.32, 1–20
Follow‐up (in years, mean ± SD)	7.60 ± 7.29	4.60 ± 6.47
Age at symptom onset (mean ± SD, median)	27.31 ± 12.28, 26	41.13 ± 11.41, 40
*n* patients with AI	150	—
*n* patients > 18 years with onset of cerebral disease during follow‐ups	52	—
Prospective cohort		
*n* patients	140	78
Visits per patient (mean ± SD, min–max)	7.13 ± 5.86, 1–23	2.59 ± 3.19, 1–20
Follow‐up (in years, mean ± SD)	7.74 ± 7.84	3.81 ± 6.42
*n* patients > 18 years with cerebral conversion during follow‐ups	25	—
*n* patients with AI	88	—
Age at symptom onset (mean ± SD, median)	27.49 ± 14.61, 26	40.85 ± 12.00, 40
AI	23.71 ± 11.76, 24	
No AI	33.67 ± 16.70, 32	
Age at inclusion visit (mean ± SD, median)	33.81 ± 14.44, 31	50.52 ± 12.44, 51
AI	30.63 ± 11.45, 29	
No AI	37.54 ± 15.12, 35	
Mean EDSS at inclusion visit (mean ± SD)	2.90 ± 1.97	3.10 ± 1.78
AI	2.65 ± 2.07	
No AI	3.56 ± 1.95	
Mean AACS at inclusion visit (mean ± SD)	4.22 ± 4.18	4.21 ± 2.97
AI	4.14 ± 4.63	
No AI	4.33 ± 3.62	

Abbreviations: AI = adrenal insufficiency, SD = standard deviation.

To investigate the prevalence of disease manifestations, only prospectively included patients were analyzed (prospective cohort, Table 1). 88 male patients (62.85%) were diagnosed with AI. Patients in this group were significantly younger when visiting the outpatient clinic for the first time (*p* < 0.001) and were younger at neurological symptom onset compared to male patients without AI (*p* = 0.002, Table 1). In the prospective cohort, 25 male patients (17.86%) developed adult‐onset cALD. The majority of cerebral conversions occurred between the ages of 30 and 40 (median 34, range: 22–67 years; Figure 1C). Out of those 25 male patients with cALD 14 male patients transitioned to arrested cALD during follow‐up; 12 patients (85.7%) after they underwent stem cell transplantation. Thirty one patients in the prospective cohort (*n* = 140; 22.1%) were classified as having arrested cALD at their first visit. Among these patients, 18 (58.1%) had received stem cell transplantation prior to their initial assessment, the remainder displayed arrest without prior treatment.

### 4.2 Disease Progression in AMN

Male patients had a mean age of AMN onset of 27 (±12.28, *n* = 140) years while females displayed a mean age of AMN onset of 41 years (±11.41, *n* = 78; Figure 1D). While women were significantly older at the time of their first visit, the severity of symptoms at first presentation was similar (*p* = 0.64, Table 1). To determine the clinical progression of AMN in adult X‐ALD patients, we specifically excluded patients with progressive and arrested cerebral disease as well as patients with Addison‐only manifestation from our analysis (145 remaining patients (*n*_male_ = 89) and 491 records (*n*_male_ = 393), Table S3). The mean increase in EDSS per year was 0.19 ± 0.30 in women and 0.34 ± 0.46 in men (Figure 1E). The mean increase in total AACS per year was 0.52 ± 0.95 for females and 0.54 ± 0.53 for males. Increase in AACS and EDSS were correlated (*ρ* = 0.78, *p* < 0.001, Figure S1B). Males reached a 50% probability of attaining an EDSS of 6, indicating severe gait disturbances by age 41 and females by age 70 (Figure 1F). Patients with an EDSS ≤ 4.5 showed a higher annual average EDSS progression rate (males 0.34 ± 0.77, females 0.11 ± 0.11) than patients with an EDSS > 4.5 (males 0.23 ± 0.33, females 0.09 ± 0.10).

### 4.3 Analysis of Very Long Chain Fatty Acids

**FIGURE 2:**
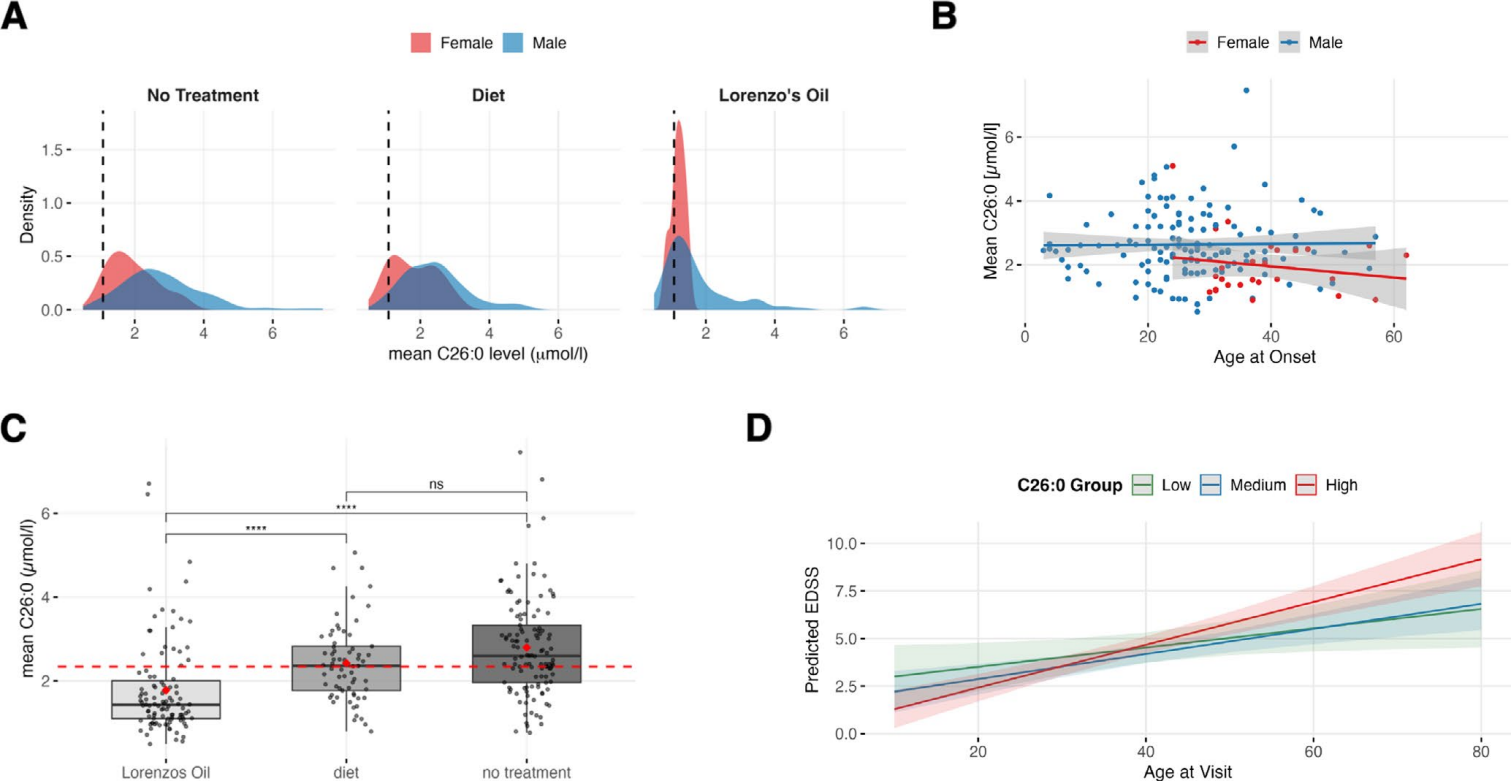
(A) Density Plot of C26:0 levels stratified by sex and nutritional treatment. Each curve reflects the distribution of values within a group (male vs. female). The black dashed line represents the cut‐off value of 1.08 μmol/L for C26:0. (B) Untreated patients' mean C26:0 levels were not associated with the age of symptom onset (male *ρ* = 0.013, *p* = 0.884; female *ρ* = −0.179, *p* = 0.371). (C) Effect of diet and monounsaturated fatty acids on plasma C26:0 (mean patient values). The red dotted line represents the mean C26:0 across the complete cohort. No treatment (Mean ± SD): 2.8 ± 1.17 μmol/L; diet: 2.42 ± 0.91; Lorenzo's Oil: 1.78 ± 1.11. Adjusted *p* values of groupwise comparisons: No treatment—diet *p* = 0.106, no treatment—Lorenzo's Oil: *p* < 0.001, diet—Lorenzo's Oil *p* = < 0.001. *p* values were corrected for multiple comparisons using Bonferroni correction. (D) Predicted EDSS based on age at visit in untreated patients with low mean C26:0 (< 1.5 μmol/L); medium mean C26:0 (1.5–2.5 μmol/L) and high mean C26:0 (> 2.5 μmol/L; *p* (interaction effect of age at visit and high mean C26:0) = 0.024).

Plasma levels of VLCFA were available from 202 male and 37 female patients in a total of 966 visits (*n*_male_ = 851, full cohort). VLCFA plasma levels in males were higher than in females (C26:0; *p* = 0.015, Figure 2A, Table 2). Values not influenced by patients' intake of monounsaturated fatty acids (e.g., Lorenzo's Oil) were available from 165 male (*n*_visits_ = 453) and 36 female patients (*n*_visits_ = 83). Due to limited sample size, further analyses were focused on male patients. In male patients, we detected significant reductions in patient‐wise mean C26:0 by dietary measures and by intake of Lorenzo's Oil compared to no treatment (Figure 2C, *p* < 0.001). However, consistent normalization was rarely reached (6.79% of patients with normalized C26:0 in all measurements, 8.74% of patients with normalized values in at least 80% of their measurements, Figure S2H). Equal effects were detected for C24:0 and C22:0 (Table 2 and Figure S2B,C,E,F). We did not find a relevant correlation of patients' mean untreated C26:0 plasma levels and age at symptom onset (male *ρ* = 0.013, *p* = 0.884; female *ρ* = −0.179, *p* = 0.371; Figure 2B). To investigate whether the combined effects of age and plasma VLCFA levels in untreated patients influence clinical progression, we employed a linear mixed‐effects model accounting for repeated visits per patient. Using a patient‐based approach, we grouped individuals based on their mean C26:0 values (low (< 1.5 μmol/L), medium (1.5–2.5 μmol/L), and high (> 2.5 μmol/L)). Applying this strategy, we detected a significant effect for the interaction of age and high mean C26:0 (*p* = 0.024, Figures 2D and S3B).

**TABLE 2:** Average VLCFA values based on mean patient values (full cohort).

	C26:0 in μmol/l	C24:0 in μmol/l	C22:0 in μmol/l
Full cohort			
All patients			
Male (mean ± SD)	2.31 ± 1.15	57.08 ± 26.28	38.3 ± 15.36
Female (mean ± SD)	1.84 ± 0.83	57.28 ± 20.76	44.47 ± 13.88
Patients without Lorenzos Oil			
Male (mean ± SD)	2.54 ± 1.1	64.14 ± 23.6	45.62 ± 13.73
Female (mean ± SD)	1.91 ± 0.84	59.55 ± 20.16	41.39 ± 15.5
Male cohort			
Dietary Interventions			
Untreated (mean ± SD)	2.8 ± 1.17	68.49 ± 24.69	43.99 ± 17.92
Diet (mean ± SD)	2.42 ± 0.91	62.66 ± 18.31	40.68 ± 11.25
Lorenzos Oil (mean ± SD)	1.78 ± 1.11	41.21 ± 25.14	31.34 ± 12.56
Adrenal Insufficiency			
AI (mean ± SD)	2.54 ± 1.12	59.36 ± 25.66	39.04 ± 14.77
No AI (mean ± SD)	2.06 ± 0.93	53.83 ± 22.50	37.53 ± 11.92

*Note:* Patients without Lorenzos Oil includes patients without lipidmodyfing treatment and patients following a VLCFA‐reduced diet.

Abbreviation: SD = standard deviation.

### 4.4 Analysis of VLCFA Based on Disease Manifestations

**FIGURE 3:**
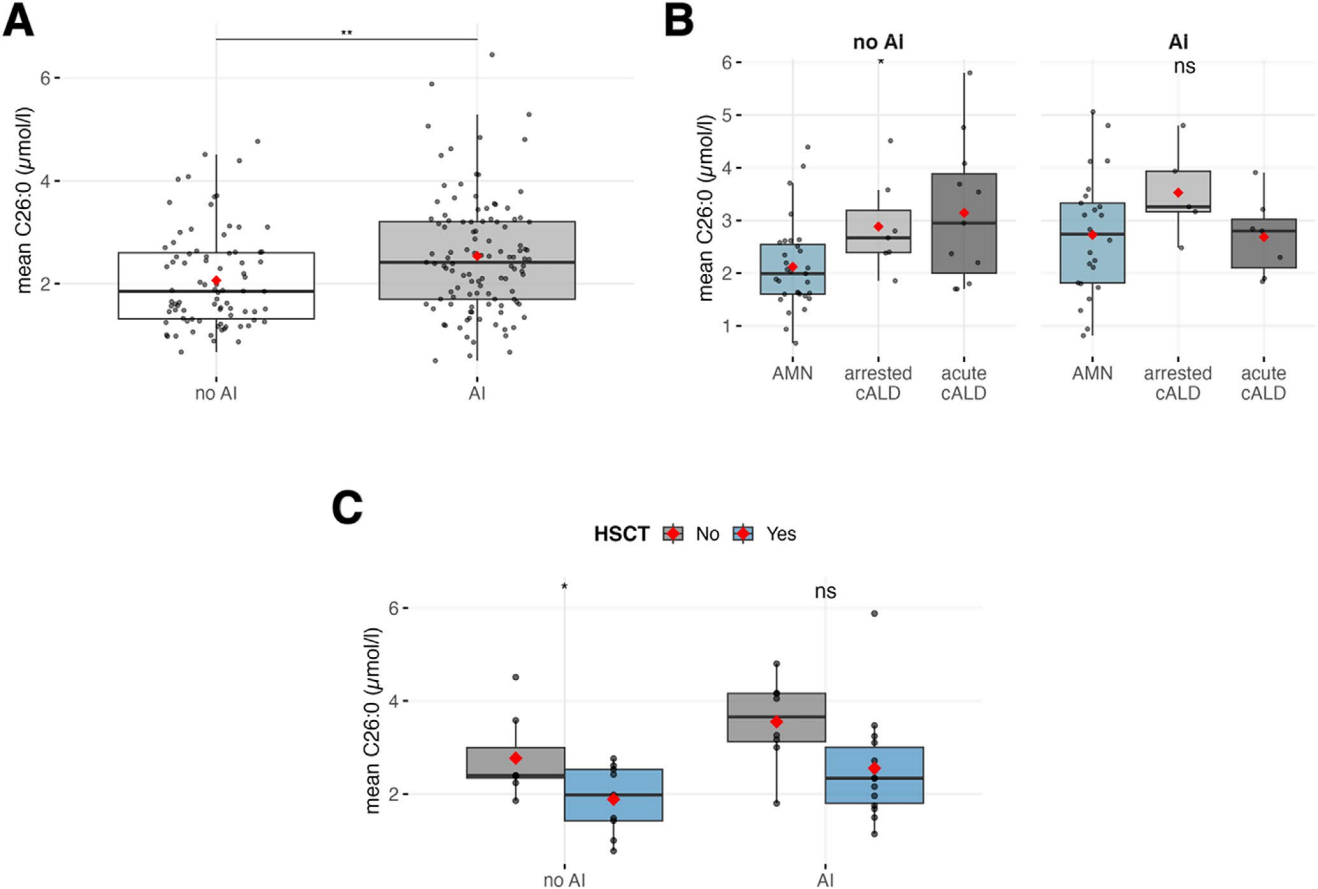
(A) Comparison of C26:0 in patients with AI versus non‐AI based on patients' average C26:0: No AI: 2.06 ± 0.93, AI: 2.54 ± 1.13, *p* = 0.002. (B) Comparison of mean C26:0 plasma values in untreated patients with AMN, arrested cALD (no transplant) and acute cALD (progressive and first detection of cerebral ALD) split according to the occurrence of AI. No AI/AMN: 2.12 ± 0.83, No AI/arrested cALD: 2.89 ± 0.89, No AI/acute cALD: 3.14 ± 1.36, *p* = 0.013 (Posthoc groupwise comparison: AMN vs. arrested cALD *p* = 0.079, AMN vs. acute cALD *p* = 0.052, arrested cALD vs. acute cALD *p* = 1.0); AI/AMN: 2.72 ± 1.12, AI/arrested cALD: 3.53 ± 0.88, AI/acute cALD: 2.68 ± 0.74, *p* = 0.25 (Posthoc groupwise comparison: AMN vs. arrested cALD *p* = 0.310, AMN vs. acute cALD *p* = 1.0, arrested cALD vs. acute cALD *p* = 0.497). (C) Comparison of mean C26:0 values of patients with arrested cALD after HSCT or spontaneously arrested stratified according to the presence of AI: No AI/noHSCT: 2.77 ± 0.86, no AI/HSCT: 1.89 ± 0.74, *p* = 0.038; AI/no HSCT: 3.55 ± 0.94 vs. AI/HSCT 2.55 ± 1.17, *p* = 0.054.

On average, C26:0 plasma levels in patients diagnosed with AI were significantly higher than in patients without AI (*p* = 0.002, Figure 3A, Table 2). This finding could also be confirmed in subgroups with and without lipid‐modifying treatments (Figure S2A, not all VCLFA species reaching significance levels, Figure S2D,G).

In non‐AI patients, VLCFA levels were significantly elevated in arrested and acute cALD compared to AMN (*p* = 0.013, Figures 3B and S3C) but post hoc groupwise comparisons did not reach statistical significance (AMN vs. arrested cALD: *p* = 0.079, AMN vs. acute cALD: *p* = 0.052, arrested cALD vs. acute cALD: *p* = 1.0). In patients with concurrent diagnosis of AI, levels of VLCFA (though highly variable) on average were equal between AMN and acute cALD.

When comparing mean VLCFA values of untreated patients with spontaneous arrested cALD with cALD who arrested after a stem cell transplant, we found significantly lower plasma levels of C26:0 in transplanted patients (Mean μmol/l ± SD; transplanted: 2.29 ± 1.06, not transplanted: 3.16 ± 0.96, *p* = 0.013). This effect remained when controlled for AI (Figure 3C). Comparisons between transplanted patients and the entire male cohort showed similar patterns (*p* = 0.014). When controlled for AI, a trend towards significance did not reach the conventional threshold (no AI/HSCT vs. no AI/no HSCT: *p* = 0.071; AI/HSCT vs. AI/no HSCT: *p* = 0.06, data not shown).

## 5 Discussion

X‐ALD is the most common hereditary leukodystrophy, often associated with a significantly reduced life span in males and substantial disease burden in both male and female adult patients due to AMN. More than 30 years after the discovery of the causative gene [1], a comprehensive understanding of the natural history of X‐ALD and, in particular, the ability to predict disease severity remains a critical and unresolved challenge. Recent findings indicate a potential association between clinical phenotype and VLCFA levels, both measured as C26:0‐lysophosphatidylcholine (C26:0 LPC) and total C26:0 concentrations, challenging the longstanding assumption of no such correlation [12]. Here, we present one of the largest single‐center cohort studies in patients with adulthood X‐ALD, providing information about the frequency of disease manifestations, progression rate, and association of phenotypes with plasma VLCFA levels.

In our cohort, male patients diagnosed with AI showed significantly higher VLCFA concentrations compared to those without this manifestation. Whether this finding indicates a causal relationship between specific genotypes, VLCFA levels, and damage to adrenal glands, or may in part be explained by treatment‐related factors, such as an external, not entirely physiologic glucocorticoid provision in AI, is an open question. Experimental evidence indicates that glucocorticoids upregulate the generation of substrates of VLCFA biosynthesis [24, 25, 26].

When assessing the combined effects of age and VLCFA levels on EDSS severity in untreated male AMN patients, we found significant combined effects indicating that high levels of C26:0 are associated with increased disease burden at higher ages. However, considering the size of our cohort, the effect is not very large. Therefore, clinical relevance for the individual patient must be interpreted with caution. We also found a trend but no significant association between VLCFA plasma levels and cerebral disease. As the occurrence of cALD in adulthood is less frequent than AI, larger multicenter studies might be required to comprehensively assess the correlation of VLCFA levels and cerebral disease [12].

In patients with cerebral involvement who had undergone HSCT, we observed a significant reduction—but not full normalization—of plasma VLCFA levels compared to patients with spontaneously arrested cerebral disease or no cerebral manifestation. Given the above‐mentioned association of long‐term disease burden and VLCFA levels, this raises the question of whether HSCT may also slow down the clinical progression of myeloneuropathic symptoms in affected males. With an increasing number of young adults who received HSCT during childhood, careful long‐term follow‐up will help to assess the emergence and trajectory of myeloneuropathic features in comparison to the natural course of untreated X‐ALD.

Typically, 15%–20% of female patients are said to have C26:0 plasma levels within the normal range [27]. Consistent with that, in our cohort more than 26% of analyzed females exhibited at least one normal measurement below cut‐off. However, only 2.63% of female patients consistently showed normal C26:0 levels across all visits. Thus, VLCFA measurements in females could provide results with high diagnostic sensitivity, if performed repeatedly. Importantly, as this is a pure adult cohort, implications on the influence of VLCFA on clinical phenotype in childhood might be different. An additional important limitation of our study is that we relied exclusively on plasma VLCFA measurements. C26:0‐LPC was postulated to be more sensitive in the diagnosis of X‐ALD [28], and in addition, C26:0 levels can be influenced by dietary intake [29]. In clinical practice, this effect is typically minimized by collecting blood samples in a fasted state. Nonetheless, occasional non‐fasted samples may occur due to patient non‐compliance or clinical routine constraints. Notably, such dietary effects have not been demonstrated for C26:0‐LPC.

This cohort is characterized by a large proportion of patients using a lipid‐modifying diet or monounsaturated fatty acids to lower their VLCFA plasma levels. Our data do not provide sufficient evidence to make a clear recommendation either in favor of or against a lipid‐modifying diet or the use of monounsaturated fatty acids. Importantly, although treatment led to a measurable reduction in plasma VLCFA levels, complete normalization was rarely achieved. This limits the interpretability of our findings and underscores the challenges posed by restricted clinical monitoring, variable patient adherence, and the need for highly individualized dosing strategies in investigating the effects of using monounsaturated fatty acids.

In addition to a thorough evaluation of the predictive value of VLCFA, our study provides one of the most comprehensive natural history datasets in adult X‐ALD. The observed median time to AMN onset and annual clinical progression rates are in line with previous publications [7, 8, 9, 30, 31]. Interestingly, the severity of symptoms that led to the presentation of the patients according to EDSS was comparable between male and female patients, but on average presentation occurred approximately 15 years later in females. Our dataset revealed AI to be present in 63% of the male patients, aligning with previous studies [8, 9]. Cerebral manifestations peaked between 30 and 40 years but occurred up to the age of 67 and in patients with late and minimal AMN burden, highlighting the importance of genetic counseling and evaluation of male asymptomatic relatives of mutation carriers, regardless of age.

The number of patients in this study who had received their diagnosis before the onset of any manifestations of disease due to genetic counseling was relatively low (17.6%). The onset of AMN in most patients was thus assessed by exploring the reported medical history and available clinical and electrophysiological data, potentially misdating the neurological onset. Routine clinical follow‐up might reveal early clinical manifestations not restricting the patients' daily activities. The availability of newborn screening will likely result in an increased inclusion of such asymptomatic and early symptomatic patients in countries where it is available. This will possibly further increase the capture of early‐stage symptoms and advance our knowledge of natural history [32]. In line with that, in patients that were diagnosed due to the presence of AI in childhood, the onset of AMN occurred significantly earlier, likely due to the earlier presentation and more continuous clinical follow‐up in the initial stages. For such patients at early disease stages and with minimal disease burden, a recent natural history study [32] suggested specifically slow progression rates. Interestingly, setting a higher cutoff (≤ 4.5 vs. ≤ 2.5), we detected a faster progression in the lower EDSS ranges. As it has been shown that increases in EDSS scores in the lower range reflect less clinically meaningful progression compared to similar increases in the higher EDSS ranges [33], this observation in our cohort is indicative of a more continuous clinical deterioration over the course of the disease, once moderate disease burden commenced. Functional tests such as posturography, which measure body sway, have been shown to be more sensitive indicators of disease progression [34, 35] and may offer a more continuous parameter than conventional clinical scores, making them valuable tools for routine clinical practice.

## 6 Conclusion

In summary, in this single‐center mixed retrospective/prospective observational study we find a significant association of AI with VLCFA levels. Though large interindividual variability exists, the data highlight the need to control for untreated VLCFA levels in studies focusing on VLCFA modulation. Clinical characteristics, especially adrenal insufficiency, should likewise be considered. In contrast to studies using C26:0‐LPC, we found no significant correlation of VLCFA levels with the presence of cALD and only weak evidence of an association with a more severe clinical burden of AMN. However, the sample size was limited for specific subgroups, highlighting the urgent need for large‐scale international studies to enable robust conclusions regarding the relationship between VLCFA levels, clinical progression, and specific manifestations of the disease. Given the considerable variability and small effect sizes, the predictive value of VLCFA levels for individual patients should be interpreted with caution.

## Author Contributions

J.L. performed the analysis. J.L. and C.B. jointly planned the analysis and co‐authored the first draft of the manuscript. J.L., M.P., F.K., L.S., W.K., and C.B. were involved in patient care at the outpatient clinic and contributed to data collection for the registry. D.P. and R.A.J. were responsible for the genetic analysis at our center and for critically reviewing all available genetic data. C.M. developed the technical framework and provided guidance and support in establishing the registry, tailoring it to the specific requirements of the leukodystrophy registry. F.K., J.J., and J.O. analyzed plasma VLCFA levels and offered technical expertise. All authors critically reviewed and revised the manuscript.

## Funding

This work was supported by Faculty of Medicine Leipzig, Clinician Scientist Programme; Deutsche Forschungsgemeinschaft, SPP2395; Sächsische Aufbaubank, 100601615; and Bundesministerium für Gesundheit, ZMVI1‐2520DAT94B.

## Conflicts of Interest

The authors declare no conflicts of interest.

## Supporting information


**Table S1:** List of R Packages Used in Analysis.

**Table S2:** Genetic information of the Leipzig X‐ALD cohort.

**Table S3:** Demographic and clinical characteristics of prospective AMN cohort.

**Figure S1:** (A) Time from first symptom onset to clinical diagnosis in male and female patients (full cohort), Negative values represent diagnoses made before symptom onset, typically in patients identified through family screening. (B) Manifestation at first and last visit of male patients in full cohort (*n* patient at each instance = 241). (C) Correlation of Increase in EDSS and AACS Score (*r* = 0.78, *p* = < 0.001, prospective cohort).
**Figure S2:** (A) Mean C26:0 levels (μmol/L) in male patients stratified by the presence or absence of adrenal insufficiency across treatment groups; red points indicate group means. No treatment/no AI: 2.30 ± 0.95, no treatment/AI: 2.79 ± 1.04, *p* = 0.018; diet/no AI: 2.24 ± 1.21, diet/AI: 2.93 ± 1.03, *p* = 0.37; Lorenzos Oil/no AI: 1.50 ± 0.61, Lorenzos Oil/AI: 2.14 ± 1.15, *p* = 0.005. Post Hoc Analysis using Dunn's Test. (B) Mean C26:0/C22:0 ratio in male patients by treatment group; red dotted line represents the overall cohort mean. No treatment: 0.07 ± 0.02; diet: 0.06 ± 0.02, Lorenzos Oil: 0.06 ± 0.03. (C) Mean C24:0 plasma levels (μmol/l) in male patients by treatment group; red dotted line indicates the cohort mean. No treatment: 68.49 ± 24.69, diet: 62.66 ± 18.31, Lorenzos Oil: 41.21 ± 25.14. (D) Mean C24:0 levels (μmol/l) in male patients based on adrenal insufficiency status across treatment groups; red points denote group means. No treatment/no AI (μmol/l): 63.39 ± 20.25, no treatment/AI: 62.12 ± 20.10, *p* = 0.91; diet/no AI: 54.54 ± 10.55, diet/AI: 73.71 ± 25.28, *p* = 0.23; Lorenzos Oil/no AI: 34.66 ± 13.61, Lorenzos Oil/AI 51.73 ± 28.78, *p* = 0.012. Post Hoc Analysis using Dunn's Test. (E) Mean C24:0/C22:0 ratio in male patients by treatment group; red dotted line indicates the cohort mean. No treatment: 1.58 ± 0.27, diet: 1.56 ± 0.24, Lorenzos Oil: 1.25 ± 0.36. (F) Mean C22:0 plasma levels (μmol/l) in male patients by treatment group; red dotted line indicates the cohort mean. No treatment: 43.99 ± 17.92, diet: 40.68 ± 11.25, Lorenzos Oil: 31.34 ± 12.56. (G) Mean C22:0 levels (μmol/L) in male patients stratified by the presence of adrenal insufficiency across treatment groups; red points denote group means. No treatment/no AI (μmol/l): 42.00 ± 10.85, no treatment/AI: 40.64 ± 12.83, *p* = 0.36; diet/no AI: 34.52 ± 2.95, diet/AI: 46.71 ± 17.16, *p* = 0.23; Lorenzos Oil/no AI: 28.83 ± 9.37, Lorenzos Oil/AI 34.84 ± 14.86, *p* = 0.11. Post Hoc Analysis using Dunn's Test. (H) Distribution of individual C26:0 levels in patients using Lorenzos Oil versus without any nutritional treatment at every available visit. Black dashed line represents cut off value of 1.08 μmol/L.
**Figure S3:** (A) Mean C24:0 in μmol/l in arrested cALD patients without nutritional treatment receiving HSCT or spontaneously arrested based on occurrence of AI or not. No AI/noHSCT: 70.26 ± 14.39, no AI/HSCT 56.67 ± 13.03, *p* = 0.058; AI/no HSCT: 69.57 ± 8.47, AI/HSCT 64.02 ± 19.16, *p* = 0.45. (B) Fixed effects of Linear mixed model analyzing EDSS progression by age based on low (< 1.5 μmol/L), medium (1.5–2.5 μmol/L) or high mean C26:0 (> 2.5 μmol/L) in male patients. (C) Mean C26:0 in μmol/l split by the presence or absence of AI and cALD (untreated): No AI/no cALD: 2.45 ± 1.01, No AI/cALD: 3.26 ± 1.68, *p* = 0.09; AI/no cALD: 22.92 ± 0.99, AI/cALD: 3.13 ± 1.03, *p* = 0.41. (D) Predicted EDSS based on age at visit in patients treated with Lorenzo's Oil with low mean C26:0 (< 1.5 μmol/L); medium mean C26:0 (1.5–2.5 μmol/L) and high mean C26:0 (> 2.5 μmol/L; *p* (interaction effect of age at visit and high mean C26:0) = 0.041). (E) Confidence Intervals of fixed effects of the linear mixed model analyzing the effects of mean patients' plasma VLCFA and age on EDSS progression in male patients treated with Lorenzo's Oil.

## Data Availability

The data that support the findings of this study are available on request from the corresponding author. The data are not publicly available due to privacy or ethical restrictions.

## References

[1] J. Mosser , A. M. Douar , C. O. Sarde , et al., “Putative X‐Linked Adrenoleukodystrophy Gene Shares Unexpected Homology With ABC Transporters,” Nature 361 (1993): 726–730, 10.1038/361726a0. 8441467

[2] I. Singh , A. E. Moser , H. W. Moser , and Y. Kishimoto , “Adrenoleukodystrophy: Impaired Oxidation of Very Long Chain Fatty Acids in White Blood Cells, Cultured Skin Fibroblasts, and Amniocytes,” Pediatric Research 18 (1984): 286–290, 10.1203/00006450-198403000-00016. 6728562

[3] J. M. Powers , D. P. DeCiero , M. Ito , A. B. Moser , and H. W. Moser , “Adrenomyeloneuropathy: A Neuropathologic Review Featuring Its Noninflammatory Myelopathy,” Journal of Neuropathology and Experimental Neurology 59 (2000): 89–102, 10.1093/jnen/59.2.89. 10749098

[4] I. C. Huffnagel , W. J. C. van Ballegoij , B. M. van Geel , J. M. B. W. Vos , S. Kemp , and M. Engelen , “Progression of Myelopathy in Males With Adrenoleukodystrophy: Towsards Clinical Trial Readiness,” Brain 142 (2019): 334–343, 10.1093/brain/awy299. 30535170

[5] M. Engelen , S. Kemp , and B.‐T. Poll‐The , “X‐Linked Adrenoleukodystrophy: Pathogenesis and Treatment,” Current Neurology and Neuroscience Reports 14 (2014): 486, 10.1007/s11910-014-0486-0. 25115486

[6] L. Schäfer , H. Roicke , C.‐C. Bergner , and W. Köhler , “Self‐Reported Quality of Life in Symptomatic and Asymptomatic Women With X‐Linked Adrenoleukodystrophy,” Brain and Behavior: A Cognitive Neuroscience Perspective 13 (2023): e2878, 10.1002/brb3.2878. PMC10013936 36748403

[7] N. R. Grant , Y. Li , L. de La Rosa Abreu , et al., “Disease Burden in Female Patients With X‐Linked Adrenoleukodystrophy,” Neurology 104 (2025): e213370, 10.1212/WNL.0000000000213370. 39919255 PMC11810135

[8] H. W. Moser , A. B. Moser , S. Naidu , and A. Bergin , “Clinical Aspects of Adrenoleukodystrophy and Adrenomyeloneuropathy,” Developmental Neuroscience 13 (1991): 254–261, 10.1159/000112170. 1817030

[9] M. de Beer , M. Engelen , and B. M. van Geel , “Frequent Occurrence of Cerebral Demyelination in Adrenomyeloneuropathy,” Neurology 83 (2014): 2227–2231, 10.1212/WNL.0000000000001074. 25378668

[10] C. G. Bergner , F. van der Meer , A. Winkler , et al., “Microglia Damage Precedes Major Myelin Breakdown in X‐Linked Adrenoleukodystrophy and Metachromatic Leukodystrophy,” Glia 67 (2019): 1196–1209, 10.1002/glia.23598. 30980503 PMC6594046

[11] M. Engelen , S. Kemp , M. de Visser , et al., “X‐Linked Adrenoleukodystrophy (X‐ALD): Clinical Presentation and Guidelines for Diagnosis, Follow‐Up and Management,” Orphanet Journal of Rare Diseases 7 (2012): 51, 10.1186/1750-1172-7-51. 22889154 PMC3503704

[12] Y. R. J. Jaspers , H. A. F. Yska , C. G. Bergner , et al., “Lipidomic Biomarkers in Plasma Correlate With Disease Severity in Adrenoleukodystrophy,” Communications Medicine 4 (2024): 175, 10.1038/s43856-024-00605-9. 39256476 PMC11387402

[13] J. López‐Erauskin , J. Galino , M. Ruiz , et al., “Impaired Mitochondrial Oxidative Phosphorylation in the Peroxisomal Disease X‐Linked Adrenoleukodystrophy,” Human Molecular Genetics 22, no. 16 (2013): 3296–3305, 10.1093/hmg/ddt186. 23604518

[14] J. Zhou , M. R. Terluk , P. J. Orchard , J. C. Cloyd , and R. V. Kartha , “N‐Acetylcysteine Reverses the Mitochondrial Dysfunction Induced by Very Long‐Chain Fatty Acids in Murine Oligodendrocyte Model of Adrenoleukodystrophy,” Biomedicine 9, no. 12 (2021): 1826, 10.3390/biomedicines9121826. PMC8698433 34944641

[15] S. Hein , P. Schönfeld , S. Kahlert , and G. Reiser , “Toxic Effects of X‐Linked Adrenoleukodystrophy‐Associated, Very Long Chain Fatty Acids on Glial Cells and Neurons From Rat Hippocampus in Culture,” Human Molecular Genetics 17, no. 12 (2008): 1750–1761, 10.1093/hmg/ddn066. 18344355

[16] H. Ali , M. Kobayashi , K. Morito , et al., “Peroxisomes Attenuate Cytotoxicity of Very Long‐Chain Fatty Acids,” Biochimica et Biophysica Acta (BBA)—Molecular and Cell Biology of Lipids 1868, no. 2 (2023): 159259, 10.1016/j.bbalip.2022.159259. 36460260

[17] S. Kemp , J. J. Orsini , M. S. Ebberink , M. Engelen , and T. C. Lund , “VUS: Variant of Uncertain Significance or Very Unclear Situation?,” Molecular Genetics and Metabolism 140 (2023): 107678, 10.1016/j.ymgme.2023.107678. 37574344

[18] C. Videbæk , L. Melgaard , A. M. Lund , and S. W. Grønborg , “Newborn Screening for Adrenoleukodystrophy: International Experiences and Challenges,” Molecular Genetics and Metabolism 140 (2023): 107734, 10.1016/j.ymgme.2023.107734. 37979237

[19] J. Wagner , H. Schneiderheinze , M. Wurlitzer , et al., “Integration of Trusted Third Party Software Into an EDC System for Data Protection ‐ Compliant Identity Management, Consent Management and Pseudonymization in Medical Research Studies,” Studies in Health Technology and Informatics 317 (2024): 75–84, 10.3233/SHTI240840. 39234709

[20] P. A. Harris , R. Taylor , R. Thielke , J. Payne , N. Gonzalez , and J. G. Conde , “Research Electronic Data Capture (REDCap)—A Metadata‐Driven Methodology and Workflow Process for Providing Translational Research Informatics Support,” Journal of Biomedical Informatics 42 (2009): 377–381, 10.1016/j.jbi.2008.08.010. 18929686 PMC2700030

[21] P. A. Harris , R. Taylor , B. L. Minor , et al., “The REDCap Consortium: Building an International Community of Software Platform Partners,” Journal of Biomedical Informatics 95 (2019): 103208, 10.1016/j.jbi.2019.103208. 31078660 PMC7254481

[22] J. F. Kurtzke , “Rating Neurologic Impairment in Multiple Sclerosis: An Expanded Disability Status Scale (EDSS),” Neurology 33 (1983): 1444–1452, 10.1212/wnl.33.11.1444. 6685237

[23] W. Köhler and P. Sokolowski , “A New Disease‐Specific Scoring System for Adult Phenotypes of X‐Linked Adrenoleukodystrophy,” Journal of Molecular Neuroscience 13, no. 3 (1999): 247–252.

[24] A. Anzulovich , A. Mir , M. Brewer , G. Ferreyra , C. Vinson , and R. Baler , “Elovl3: A Model Gene to Dissect Homeostatic Links Between the Circadian Clock and Nutritional Status,” Journal of Lipid Research 47 (2006): 2690–2700, 10.1194/jlr.M600230-JLR200. 17003504

[25] A. Brolinson , S. Fourcade , A. Jakobsson , A. Pujol , and A. Jacobsson , “Steroid Hormones Control Circadian Elovl3 Expression in Mouse Liver,” Endocrinology 149 (2008): 3158–3166, 10.1210/en.2007-1402. 18292190

[26] R. Ofman , I. M. E. Dijkstra , C. W. T. van Roermund , et al., “The Role of ELOVL1 in Very Long‐Chain Fatty Acid Homeostasis and X‐Linked Adrenoleukodystrophy,” EMBO Molecular Medicine 2 (2010): 90–97, 10.1002/emmm.201000061. 20166112 PMC3377275

[27] A. B. Moser , N. Kreiter , L. Bezman , et al., “Plasma Very Long Chain Fatty Acids in 3,000 Peroxisome Disease Patients and 29,000 Controls,” Annals of Neurology 45 (1999): 100–110, 10.1002/1531-8249(199901)45:1<100:aid-art16>3.0.co;2-u. 9894883

[28] Y. R. J. Jaspers , S. Ferdinandusse , I. M. E. Dijkstra , et al., “Comparison of the Diagnostic Performance of C26:0‐Lysophosphatidylcholine and Very Long‐Chain Fatty Acids Analysis for Peroxisomal Disorders,” Frontiers in Cell and Development Biology 8 (2020): 690, 10.3389/fcell.2020.00690. PMC7438929 32903870

[29] C. Lam , D. Wong , S. Cederbaum , B. Lim , and Y. Qu , “Peanut Consumption Increases Levels of Plasma Very Long Chain Fatty Acids in Humans,” Molecular Genetics and Metabolism 107 (2012): 620–622, 10.1016/j.ymgme.2012.07.015. 22864056

[30] B. M. van Geel , L. Bezman , D. J. Loes , et al., “Evolution of Phenotypes in Adult Male Patients With X‐Linked Adrenoleukodystrophy,” Annals of Neurology 49 (2001): 186–194, 10.1002/1531-8249(20010201)49:2<186:aid-ana38>3.0.co;2-r. 11220738

[31] E. J. Mallack , S. van de Stadt , P. A. Caruso , et al., “Clinical and Radiographic Course of Arrested Cerebral Adrenoleukodystrophy,” Neurology 94 (2020): e2499–e2507, 10.1212/WNL.0000000000009626. 32482842 PMC7455338

[32] H. A. F. Yska , M. Voermans , E. Kabak , and M. Engelen , “Progression of Spinal Cord Disease in Adult Men With Adrenoleukodystrophy,” Journal of Inherited Metabolic Disease 48 (2025): e12845, 10.1002/jimd.12845. 39777696 PMC11706703

[33] J. Müller , A. Cagol , J. Lorscheider , et al., “Harmonizing Definitions for Progression Independent of Relapse Activity in Multiple Sclerosis: A Systematic Review,” JAMA Neurology 80 (2023): 1232–1245, 10.1001/jamaneurol.2023.3331. 37782515

[34] J. L. Keller , A. Eloyan , G. V. Raymond , A. Fatemi , and K. M. Zackowski , “Sensorimotor Outcomes in Adrenomyeloneuropathy Show Significant Disease Progression,” Journal of Inherited Metabolic Disease 45, no. 2 (2022): 308–317, 10.1002/jimd.12457. 34796974 PMC8987487

[35] H. A. F. Yska , B. R. Turk , A. Fatemi , et al., “International Validation of Meaningfulness of Postural Sway and Gait to Assess Myeloneuropathy in Adults With Adrenoleukodystrophy,” Journal of Inherited Metabolic Disease 47, no. 6 (2024): 1336–1347, 10.1002/jimd.12753. 38795020 PMC11586604

