# Supplementary material for: Natural History of Clinical Phenotypes and Their Biochemical Correlates in Adult X‐Linked Adrenoleukodystrophy
Source: J Inherit Metab Dis. 2026 Mar 19;49(2):e70176. doi: 10.1002/jimd.70176 (PMC13000868; doi:10.1002/jimd.70176)
Supplement: Supplementary file 1 — Table S1: List of R Packages Used in Analysis. [file JIMD-49-0-s003.docx]

**Supplemental Table 1: List of R Packages Used in Analysis**

| R Package |
| --- |
| Broom |
| Coin |
| Emmeans |
| FSA |
| Ggeffects |
| Ggpubr |
| Lme4 |
| Lmertest |
| Lubridate |
| Readr |
| Rstatix |
| Scales |
| sjPlot |
| survival |
| survminer |
| Tidyverse |
