# Supplementary material for: Natural History of Clinical Phenotypes and Their Biochemical Correlates in Adult X‐Linked Adrenoleukodystrophy
Source: J Inherit Metab Dis. 2026 Mar 19;49(2):e70176. doi: 10.1002/jimd.70176 (PMC13000868; doi:10.1002/jimd.70176)
Supplement: Supplementary file 2 — Table S2: Genetic information of the Leipzig X‐ALD cohort. [file JIMD-49-0-s001.pdf]

Supplemental Table 2: ***Genetic information of the Leipzig X-ALD cohort***

Variant	Protein	n	listed on ClinVar	listed on ABCD1 Variant Registry	Molecular consequence	Germline classification	Classification criteria	references
c.(900-1_901+1)_(1081+1_1082-1)del	p.(Val301Metfs*5)	1	no	no	Deletion Exon 2	pathogenic	PVS1, PM2, PP4 (11 points)	PubMed: 27248780
c.10dup	p.(Leu4Profs*191)	1	yes	no	frameshift	pathogenic	PVS1, PM2, PP4 (11 points)	
c.(1082+1_1083-1)_(1488+1_1489_1)del	p.(Asp361Glyfs*59)	3	no	no	Deletion Exon 3-5	pathogenic	PVS1, PM2, PP4 (11 points)	**not yet reported**
c.1092dupC	p.(Val365Argfs*36)	1	no	no	frameshift	pathogenic	PVS1, PM2, PP4 (11 points)	original report missing
c.1156A>C	p.(Thr386Pro)	2	yes	no	missense	Uncertain significance	PM2, PP3, PP4 (4 points)	
c.1165C>T	p.(Arg389Cys)	1	yes	yes	missense	Likely pathogenic	PS4_MOD, PM2_SUP, PM5, PP3, PP4 (7 points)	PubMed: 15800013, PubMed: 22280810, PubMed: 30564185, PubMed: 32307584
c.1208T>G	p.(Met403Arg)	1	no	no	missense	Likely pathogenic	PS1, PM2, PP3, PP4 (8 points)	original report missing
c.1252C>T	p.(Arg418Trp)	8	yes	yes	missense	Pathogenic	PS4_STR, PM2_SUP, PM5_STR, PP3, PP4 (11 points)	PubMed: 39170489, PubMed: 36380532, PubMed: 36046390
c.1253G>A	p.(Arg418Gln)	1	yes	yes	missense	Likely pathogenic	PS4_MOD, PM5_STR, PP3, PP4 (8 points)	PubMed: 36256460, PubMed: 35466195
c.1386del	p.(Ile463Serfs*95)	1	no	yes	frameshift	Pathogenic	PVS1, PM2, PP4 (11 points)	
c.1390C>T	p.(Arg464Ter)	2	yes	yes	nonsense	Pathogenic	PVS1, PS2_MOD, PS4, PM2, PP4 (16 points)	PubMed: 8040304, PubMed: 34946879, PubMed: 36380532, PubMed: 40063902
c.1415_16delAG	p.(Gln472Argfs*83)	7	no	yes	frameshift	Pathogenic	PVS1, PS4, PM2, PP4 (15 points)	PubMed: 40063902, PubMed: 40256457, PubMed: 40210590, PubMed: 36925939, PubMed: 34347682
c.1426T>C	p.(Cys476Arg)	1	no	no	missense	Uncertain significance	PM2, PP3, PP4 (4 points)	**not yet reported**
c.1451C>G	p.(Pro484Arg)	1	yes	yes	missense	Pathogenic	PS4_MOD, PM2, PM5_STR, PP3, PP4 (10 points)	PubMed: 7811247, PubMed: 10551832
c.1451C>T	p.(Pro484Leu)	1	no	no	missense	Likely pathogenic	PS4_SUP, PM2, PM5_STR, PP3, PP4 (9 points)	PubMed: 36537231
c.1522C>T	p.(Pro508Ser)	1	no	yes	missense	Likely pathogenic	PM5, PM2, PP3, PP4 (6 points)	
c.1523C>T	p.(Pro508Leu)	10	yes	yes	missense	Pathogenic	PS4_STR, PM2, PM5_SUP, PP3, PP4 (11 points)	PubMed: 21300044, PubMed: 34826210
c.1527T>A	p.(Asn509Lys)	1	yes	no	missense	Likely pathogenic	PM5_STR, PM2, PP3, PP4 (8 points)	PubMed: 21300044, PubMed: 34826210
c.1528G>A	p.(Gly510Ser)	1	yes	yes	missense	Likely pathogenic	PM5, PM2, PP3, PP4 (6 points)	
c.1534G>A	p.(Gly512Ser)	4	yes	yes	missense	Pathogenic	PS4, PM2, PM5_STR, PP3, PP4 (12 points)	PubMed: 39051462, PubMed: 34826210, PubMed: 7581394
c.1552C>T	p.(Arg518Trp)	1	yes	yes	missense	Pathogenic	PS4, PM2, PM5_STR, PP3, PP4 (12 points)	
c.1553G>A	p.(Arg518Gln)	1	yes	yes	missense	Pathogenic	PS4, PS2_MOD, PM2_SUP, PM5_STR,PP3, PP4 (13 points)	PubMed: 39853971, PubMed: 34826210, PubMed: 31074578, PubMed: 16415970
c.1557C>A	p.(Ile519=)	1	no	no	synonymous	Uncertain significance	PM2 (2 points)	**not yet reported**
c.1628C>T	p.(Pro543Leu)	1	yes	yes	missense	Pathogenic	PS4_MOD, PM2, PM5_STR, PP3, PP4 (10 points)	PubMed: 33547378, PubMed: 37597066
c.163C>T	p.(Gln55*)	1	no	yes	truncation	Pathogenic	PVS1, PS4_MOD, PM2, PP4 (13 points)	PubMed: 22479560

c.1652G>C	p.(Gly551Ala)	1	no	no	missense	Uncertain significance	PM2, PP3, PP4 (4 points)	PubMed: 33920672
c.1667A>T	p.(Gln556Leu)	1	yes	no	missense	Likely pathogenic	PS4_SUP, PM2, PM5_SUP, PP3, PP4 (6	
c.1670T>G	p.(Val557Gly)	1	yes	no	missense	Uncertain significance	PS4_SUP, PM2, PP3, PP4 (5 points)	
c.1678C>T	p.(Pro560Ser)	4	yes	yes	missense	Pathogenic	PS4_MOD, PM2, PM5_STR, PP3, PP4 (10 points)	PubMed: 11748843
c.1765del	p.(Leu589Cysfs*47)	1	no	yes	frameshift	Pathogenic	PVS1, PS4_SUP, PM2, PP4 (12 points)	PubMed: 11748843
c.1772G>A	p.(Arg591Gln)	3	yes	yes	missense	Pathogenic	PS4, PM2_SUP, PM5_STR, PP3, PP4 (11 points)	PubMed: 7668254, PubMed: 10551832, PubMed: 33920672
c.1780G>A	p.(Gly594Ser)	6	no	yes	missense	Likely pathogenic	PS4_MOD, PM2, PM5_SUP, PP3, PP4 (7 points)	PubMed: 20228476
c.1780G>C	p.(Gly594Arg)	2	yes	yes	missense	Likely pathogenic	PS4_MOD, PM2, PM5_SUP, PP3, PP4 (7 points)	PubMed: 39853971
c.1792_1793delAT	p.(Met598Valfs*2)	1	no	no	frameshift	Pathogenic	PVS1, PS4_SUP, PM2, PP4 (12 points)	10.1172/JCI117363 (PubMed: 8040304)
c.1822G>A	p.(Gly608Ser)	1	yes	yes	missense	Likely pathogenic	PS4_MOD, PS2_MOD, PM2, PP3, PP4 (8 points)	PubMed: 15800013
c.1825G>A	p.(Glu609Lys)	4	yes	yes	missense	Pathogenic	PS4, PM2, PM5, PP3, PP4 (12 points)	PubMed: 39853971, PubMed: 33920672, PubMed: 33920672
c.1829A>G	p.(Lys610Arg)	2	no	no	missense	Uncertain significance	PM2, PP3, PP4 (4 points)	**not yet reported**
c.1849C>T	p.(Arg617Cys)	2	yes	yes	missense	Pathogenic	PS4, PM2_SUP, PM5_STR, PP3, PP4 (11 points)	PubMed: 8040304, PubMed: 31227335, PubMed: 34997422
c.1850G>A	p.(Arg617His)	1	yes	yes	missense	Pathogenic	PS4, PS2_MOD, PM2_SUP, PM5_STR,PP3, PP4 (13 points)	PubMed: 8040304, PubMed: 7841445, PubMed: 37586839
c.1876G>C	p.(Ala626Pro)	1	no	no	missense	Likely pathogenic	PS4_SUP, PM2, PM5_STR, PP3, PP4 (9 points)	PMID: 34826210
c.1900G>A	p.(Ala634Thr)	1	no	yes	missense	Uncertain significance	PS4_MOD, PM2_SUP, PP3, PP4 (5 points)	PubMed: 35076462, PubMed: 34946879, PubMed: 39803877
c.1933del	p.(Gln645Argfs*46)	2	yes	yes	frameshift	Pathogenic	PVS1, PS4_MOD, PM2, PP4 (13 points)	
c.1978C>T	p.(Arg660Trp)	4	yes	yes	missense	Pathogenic	PS2_MOD, PS4_MOD, PM2, PM5_STR, PP3, PP4 (12 points)	PubMed: 7825602, PubMed: 31665121, PubMed: 34012265, PubMed: 36380532
c.1979G>A	p.(Arg660Gln)	1	yes	yes	missense	Likely pathogenic	PS4_MOD, PM2_SUP, PM5_STR, PP3, PP4 (9 points)	PubMed: 21966424, PubMed: 21889498, PubMed: 34946879
c.1992-2A>C	p.?	1	yes	no	splice acceptor loss	Likely pathogenic	PVS1_MOD, PS1_SUP, PM2, PP4 (6 points)	
c.2006_2007dup	p.(Leu670Thrfs*22)	1	no	no	frameshift	Likely pathogenic	PVS1, PM2, PP4 (11 points)	**not yet reported**
c.2010dupG	p.(Leu671Alafs*63)	1	no	yes	Deleterious mutation.	Likely pathogenic	PVS1_MOD, PS4_SUP, PM2, PP4 (6 points)	
c.2030C>A	p.(Gly677Asp)	1	no	no	missense	Uncertain significance	PS4_SUP, PM2, PP3, PP4 (5 points)	PubMed: 21700483
c.234_565del	p.(Leu79Alafs*5)	1	yes	no	frameshift	Pathogenic	PVS1, PM2, PP4 (11 points)	
c.281T>C	p.(Leu94Pro)	1	no	no	missense	Uncertain significance	PM2, PP3, PP4 (4 points)	**not yet reported**
c.293C>A	p.(Ser98Ter)	2	yes	yes	nonsense	Pathogenic	PVS1, PS4_MOD, PM2, PP4 (13 points)	PubMed: 23419472
c.293C>T	p.(Ser98Leu	2	yes	yes	missense	Likely pathogenic	PS4_MOD, PM2, PM5, PP3, PP4 (8 points)	PubMed: 8651290, PubMed: 31074578, PubMed: 37586839, PubMed: 40210590
c.30G>A	p.(Trp10Ter)	1	yes	yes	nonsense	Pathogenic	PVS1, PS4_MOD, PM2, PP4 (13 points)	PubMed: 8651290
c.310C>T	p.(Arg104Cys)	2	yes	yes	missense	Pathogenic	PS4_MOD, PM2, PM5_STR, PP3, PP4 (10 points)	PubMed: 7825602, PubMed: 11102997, PubMed: 37586839

c.311G>A	p.(Arg104His)	2	yes	yes	missense	Pathogenic	PS4_MOD, PM2, PM5_STR, PP3, PP4 (10 points)	PubMed: 7717396, PubMed: 34826210, PubMed: 33920672
c.311G>T	p.(Arg104Leu)	1	no	yes	missense	Likely pathogenic	PS4_SUP, PM2_SUP, PM5_STR, PP3, PP4 (8 points)	
c.320T>C	p.(Leu107Pro)	4	yes	yes	missense	Likely pathogenic	PS4_MOD, PM2, PM5_SUP, PP3, PP4 (7 points)	PubMed: 8566952, PubMed: 31418856
c.346G>C	p.(Gly116Arg)	1	yes	yes	missense	Pathogenic	PS1, PS4_SUP, PM2, PM5, PP3, PP4 (13 points)	PubMed: 31777199, PubMed: 36380532
c.355del	p.(Ala119Profs*79)	3	no	yes	frameshift	Pathogenic	PVS1, PM2, PP4 (11 points)	
c.385dup	p.(Ala129Glyfs*66)	1	no	yes	frameshift	Pathogenic	PVS1, PM2, PP4 (11 points)	
c.421G>A	p.(Ala141Thr)	1	yes	yes	missense	Pathogenic	PS4_MOD, PM2, PM5_STR, PP3, PP4 (10 points)	PubMed: 7581394, PubMed: 31227335, PubMed: 40210590
c.428C>T	p.(Pro143Leu)	1	yes	yes	missense	Pathogenic	PS4_MOD, PM2, PM5_STR, PP3, PP4 (10 points)	
c.454C>T	p.(Arg152Cys)	8	yes	yes	missense	Likely pathogenic	PS4_MOD, PM2_SUP, PM5_STR, PP3, PP4 (9 points)	PubMed: 7581394, PubMed: 14586615, PubMed: 29557549
c.459C>G	p.(Tyr153Ter)	2	no	yes	nonsense	Pathogenic	PVS1, PS4_SUP, PM2, PP4 (12 points)	PubMed: 31069529
c.521A>G	p.(Tyr174Cys)	1	yes	yes	missense	Pathogenic	PS4_MOD, PM2, PM5_STR, PP3, PP4 (10 points)	PubMed: 10737980, PubMed: 17542813, PubMed: 39566008, PubMed: 38436991
c.524_526delTCT	p.(Phe175del)	2	no	no	in-frame deletion	Likely pathogenic	PS4_MOD, PM2, PM4, PP4 (7 points)	PubMed: 12175782
c.526_528del	p.(Ser176del)	1	no	no	in-frame deletion	Likely pathogenic	PS4_SUP, PM2, PM4, PP4 (6 points)	PubMed: 37974207
c.562_1082-1760del	p.?	1	no	no	truncation	pathogenic	PVS1, PM2, PP4 (11 points)	**not yet reported**
c.593C>G	p.(Thr198Arg)	1	no	yes	missense	pathogenic	PS4_MOD, PM2, PM5_STR, PP3, PP4 (10 points)	PubMed: 21700483, PubMed: 37180992
c.681C>A	p.(Tyr227*)	1	no	yes	nonsense	Pathogenic	PVS1, PM2, PP4 (11 points)	
c.726G>A	p.(Trp242*)	1	no	yes	nonsense	Pathogenic	PVS1, PS4_SUP, PM2, PP4 (11 points)	PubMed: 8566952
c.796G>A	p.(Gly266Arg)	1	yes	yes	missense	Pathogenic	PS2_MOD, PS4, PM2_SUP, PM5_STR, PP3, PP4 (13 points)	PubMed: 7849723, PubMed: 34826210, PubMed: 34997422, PubMed: 37981684
c.799G>A	p.(Glu267Lys)	2	yes	yes	missense	Uncertain significance	PS4_MOD, PM2, PP4 (5 points)	PubMed: 9551465
c.818C>T	p.(Ala273Val)	1	yes	yes	missense	Uncertain significance	PM2_SUP, PM5, PP3, PP4 (5 points)	PubMed: 33920672
c.828_829insAAT	p.(Lys276_Gly277insAsn)	1	no	no	in-frame insertion	Likely pathogenic	PS4_SUP, PM2, PM4_SUP, PM5_SUP, PP4 (6 points)	PubMed: 8566952
c.838C>T	p.(Arg280Cys)	1	yes	yes	missense	Likely pathogenic	PS4_MOD, PM2_SUP, PM5, PP3, PP4 (7 points)	PubMed: 15811009, PubMed: 33920672
c.839G>A	p.(Arg280His)	2	yes	yes	missense	Likely pathogenic	PS4_MOD, PM2, PM5, PP3, PP4 (8 points)	PubMed: 35076462, PubMed: 33920672, PubMed: 39803877
c.851C>T	p.(Ser284Leu)	1	yes	yes	missense	Likely pathogenic	PS4_MOD, PM2, PM5_SUP, PP3, PP4 (7 points)	PubMed: 35466195
c.887A>G	p.(Tyr296Cys)	8	yes	yes	missense	Pathogenic	PS2_MOD, PS4, PM2, PM5_STR, PP3, PP4 (14 points)	PubMed: 35645283, PubMed: 34997422, PubMed: 25275259
c.892G>T	p.(Gly298Cys)	1	no	yes	missense	Likely pathogenic	PS4_SUP, PM2, PM5_STR, PP3, PP4 (9 points)	
c.900G>A	p.[Glu300=,Glu300_Val301insValGlyGln]	1	yes	yes	synonymous variant	Likely pathogenic	PVS1_STR(RNA), PS4_MOD, PM2, PP4 (9 points)	PubMed: 11810273
c.99C>G	p.(Tyr33*)	2	no	no	truncation	Pathogenic	PVS1, PM2, PP4 (11 points)	**not yet reported**
