# Supplementary material for: Natural History of Clinical Phenotypes and Their Biochemical Correlates in Adult X‐Linked Adrenoleukodystrophy
Source: J Inherit Metab Dis. 2026 Mar 19;49(2):e70176. doi: 10.1002/jimd.70176 (PMC13000868; doi:10.1002/jimd.70176)
Supplement: Supplementary file 3 — Table S3: Demographic and clinical characteristics of prospective AMN cohort. [file JIMD-49-0-s004.docx]

**Supplemental Table 3: Demographic and clinical characteristics of prospective AMN cohort**

|  | Male | Female |
| --- | --- | --- |
| *Prospective AMN cohort* |  |  |
| n patients | 89 | 54 |
| Visits per patient (mean ± *SD*, min–max) | 4.41 ± 3.67, 1-16 | 1.75 ± 1.36, 1-7 |
| Follow-up in days | 2062.88 | 735.81 |
| n patients with AI | 53 | - |
| Age at symptom onset (mean ± *SD*, median) | 30.6 ± 12.41, 27.5 | 40.85 ± 12.00, 40.5 |
| AI | 27.41 ± 10.28, 26.0 |  |
| No AI | 35.17 ± 13.83, 32.5 |  |
| Age at visit (mean ± *SD*, median) | 37.63 ± 14.34, 35 | 53.46 ± 10.49, 53.5 |
| Mean EDSS at inclusion visit (mean ± *SD*) | 3.24 ± 1.58 | 3.60 ± 1.54 |
| AI | 2.88 ± 1.47 |  |
| No AI | 3.69 ± 1.68 |  |
| Mean AACS at inclusion visit (mean ± *SD*) | 3.53 ± 2.42 | 4.74 ± 2.87 |
| AI | 3.37 ± 2.36 |  |
| No AI | 3.86 ± 2.58 |  |

Abbreviations: AI – adrenal insufficiency, SD = standard deviation
