# Supplementary material for: Natural History of Clinical Phenotypes and Their Biochemical Correlates in Adult X‐Linked Adrenoleukodystrophy
Source: J Inherit Metab Dis. 2026 Mar 19;49(2):e70176. doi: 10.1002/jimd.70176 (PMC13000868; doi:10.1002/jimd.70176)
Supplement: Supplementary file 4 — Figure S1: (A) Time from first symptom onset to clinical diagnosis in male and female patients (full cohort), Negative values represent diagnoses made before symptom onset, typically in patients identified through family screening. (B) Manifestation at first and last visit of male patients in full cohort (n patient at each instance = 241). (C) Correlation of Increase in EDSS and AACS Score (r = 0.78, p = < 0.001, prospective cohort). Figure S2: (A) Mean C26:0 levels (μmol/L) in male patients stratified by the presence or absence of adrenal insufficiency across treatment groups; red points indicate group means. No treatment/no AI: 2.30 ± 0.95, no treatment/AI: 2.79 ± 1.04, p = 0.018; diet/no AI: 2.24 ± 1.21, diet/AI: 2.93 ± 1.03, p = 0.37; Lorenzos Oil/no AI: 1.50 ± 0.61, Lorenzos Oil/AI: 2.14 ± 1.15, p = 0.005. Post Hoc Analysis using Dunn's Test. (B) Mean C26:0/C22:0 ratio in male patients by treatment group; red dotted line represents the overall cohort mean. No treatment: 0.07 ± 0.02; diet: 0.06 ± 0.02, Lorenzos Oil: 0.06 ± 0.03. (C) Mean C24:0 plasma levels (μmol/l) in male patients by treatment group; red dotted line indicates the cohort mean. No treatment: 68.49 ± 24.69, diet: 62.66 ± 18.31, Lorenzos Oil: 41.21 ± 25.14. (D) Mean C24:0 levels (μmol/l) in male patients based on adrenal insufficiency status across treatment groups; red points denote group means. No treatment/no AI (μmol/l): 63.39 ± 20.25, no treatment/AI: 62.12 ± 20.10, p = 0.91; diet/no AI: 54.54 ± 10.55, diet/AI: 73.71 ± 25.28, p = 0.23; Lorenzos Oil/no AI: 34.66 ± 13.61, Lorenzos Oil/AI 51.73 ± 28.78, p = 0.012. Post Hoc Analysis using Dunn's Test. (E) Mean C24:0/C22:0 ratio in male patients by treatment group; red dotted line indicates the cohort mean. No treatment: 1.58 ± 0.27, diet: 1.56 ± 0.24, Lorenzos Oil: 1.25 ± 0.36. (F) Mean C22:0 plasma levels (μmol/l) in male patients by treatment group; red dotted line indicates the cohort mean. No treatment: 43.99 ± 17.92, diet: 40.68 ± 11.25, Lor [file JIMD-49-0-s002.docx]

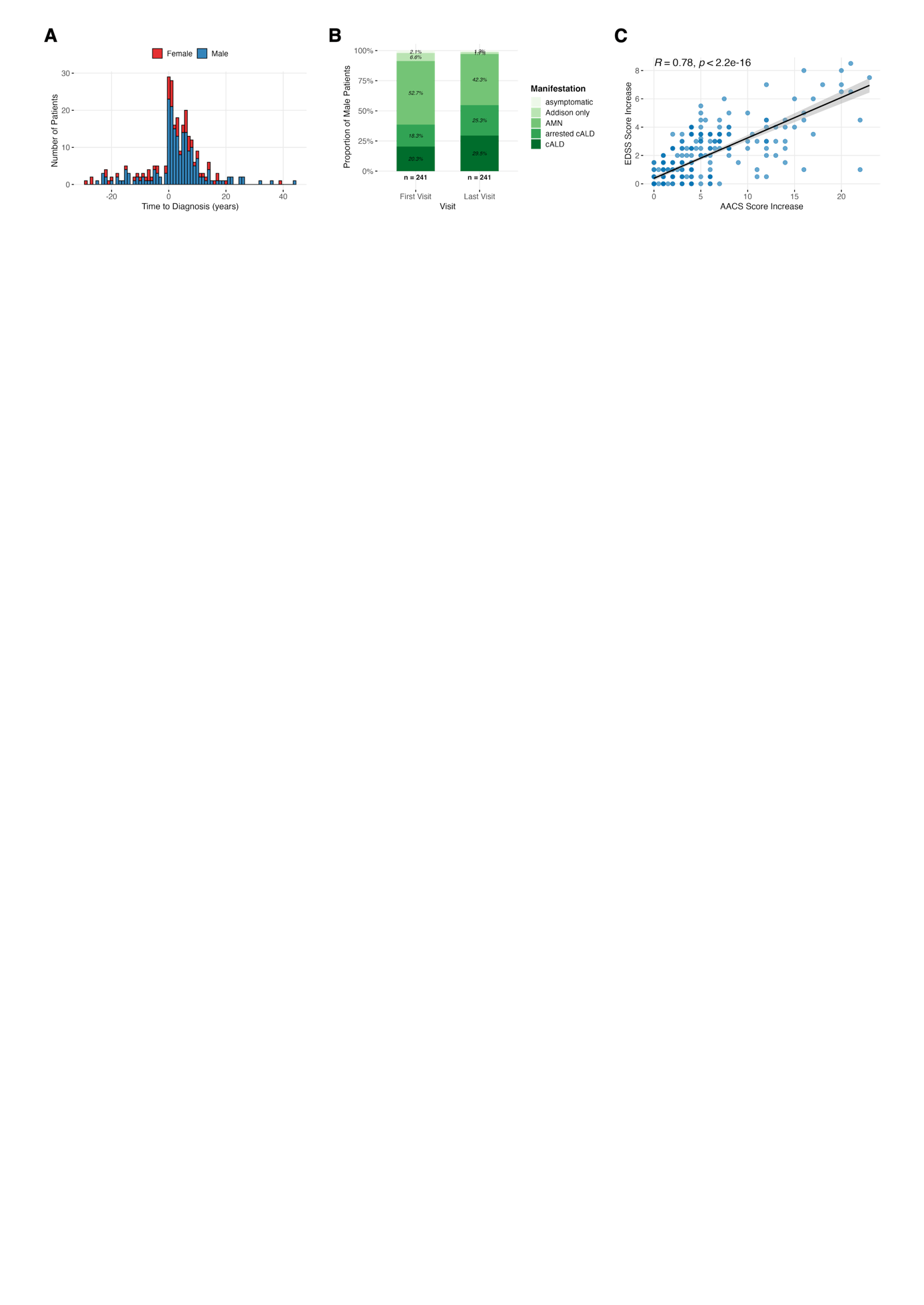


**Supplementary Figure 1:** A: Time from first symptom onset to clinical diagnosis in male and female patients (full cohort), Negative values represent diagnoses made before symptom onset, typically in patients identified through family screening. B: Manifestation at first and last visit of male patients in full cohort (n patient at each instance = 241). C: Correlation of Increase in EDSS and AACS Score (*r* = 0.78, *p*= <0.001, prospective cohort).


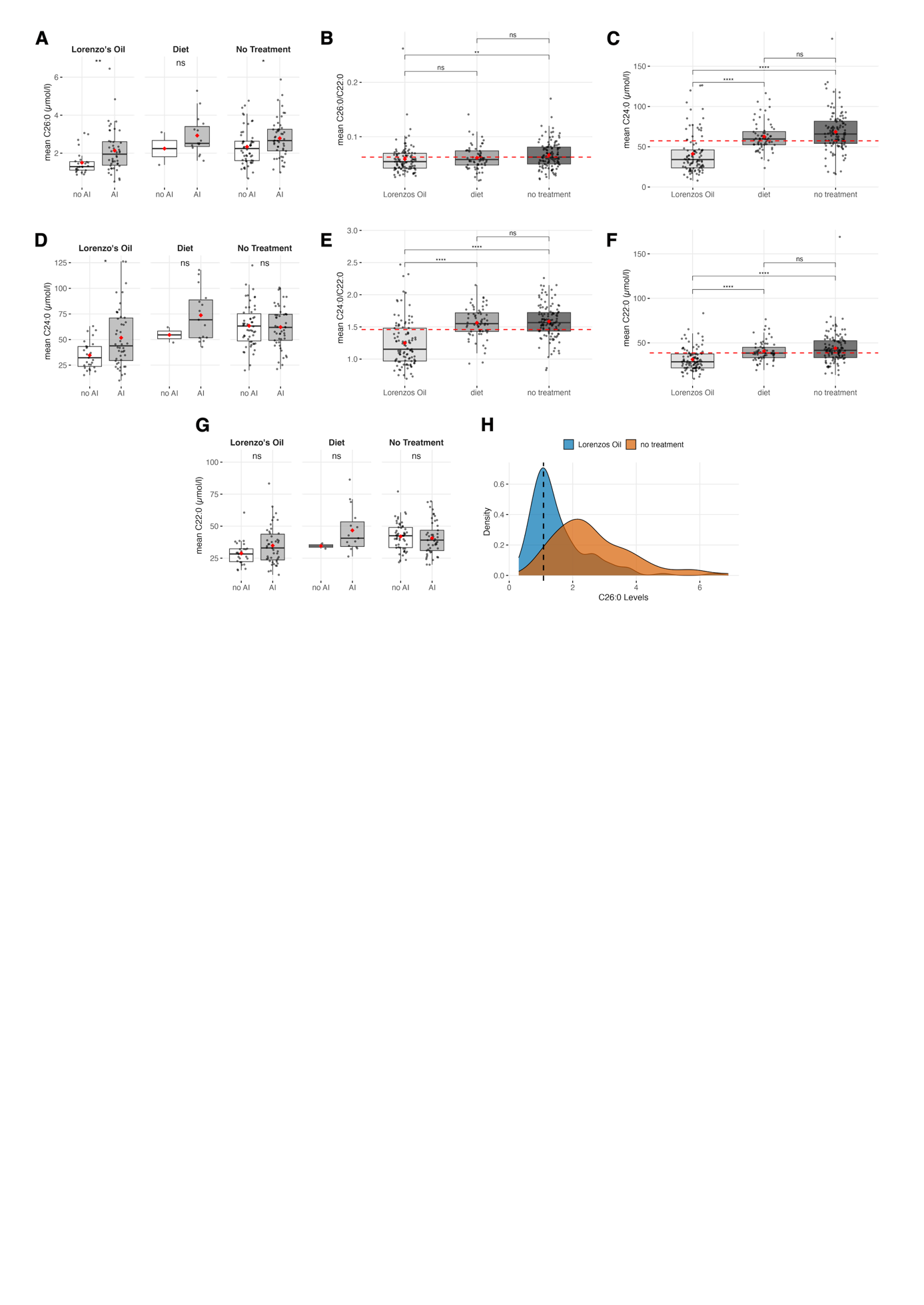


**Supplementary Figure 2:** A: Mean C26:0 levels (µmol/L) in male patients stratified by the presence or absence of adrenal insufficiency across treatment groups; red points indicate group means. No treatment/no AI: 2.30 ± 0.95, no treatment/AI: 2.79 ± 1.04, *p*= 0.018; diet/no AI: 2.24 ± 1.21, diet/AI: 2.93 ± 1.03, *p*= 0.37; Lorenzos Oil/no AI: 1.50 ± 0.61, Lorenzos Oil/AI: 2.14 ± 1.15, *p*= 0.005. Post-Hoc Analysis using Dunn’s Test. B: Mean C26:0/C22:0 ratio in male patients by treatment group; red dotted line represents the overall cohort mean. No treatment: 0.07 ± 0.02; diet: 0.06 ± 0.02, Lorenzos Oil: 0.06 ± 0.03. C: Mean C24:0 plasma levels (µmol/l) in male patients by treatment group; red dotted line indicates the cohort mean. No treatment: 68.49 ± 24.69, diet: 62.66 ± 18.31, Lorenzos Oil: 41.21 ± 25.14. D: Mean C24:0 levels (µmol/l) in male patients based on adrenal insufficiency status across treatment groups; red points denote group means. No treatment/no AI (µmol/l): 63.39 ± 20.25, no treatment/AI: 62.12 ± 20.10, *p*= 0.91; diet/no AI: 54.54 ± 10.55, diet/AI: 73.71 ± 25.28, *p*= 0.23; Lorenzos Oil/no AI: 34.66 ± 13.61, Lorenzos Oil/AI 51.73 ± 28.78, *p*= 0.012. Post-Hoc Analysis using Dunn’s Test. E: Mean C24:0/C22:0 ratio in male patients by treatment group; red dotted line indicates the cohort mean. No treatment: 1.58 ± 0.27, diet: 1.56 ± 0.24, Lorenzos Oil: 1.25 ± 0.36. F: Mean C22:0 plasma levels (µmol/l) in male patients by treatment group; red dotted line indicates the cohort mean. No treatment: 43.99 ± 17.92, diet: 40.68 ± 11.25, Lorenzos Oil: 31.34 ± 12.56. G: Mean C22:0 levels (µmol/L) in male patients stratified by the presence of adrenal insufficiency across treatment groups; red points denote group means. No treatment/no AI (µmol/l): 42.00 ± 10.85, no treatment/AI: 40.64 ± 12.83, *p*= 0.36; diet/no AI: 34.52 ± 2.95, diet/AI: 46.71 ± 17.16, *p*= 0.23; Lorenzos Oil/no AI: 28.83 ± 9.37, Lorenzos Oil/AI 34.84 ± 14.86, *p*= 0.11. Post-Hoc Analysis using Dunn’s Test. H: Distribution of individual C26:0 levels in patients using Lorenzos Oil versus without any nutritional treatment at every available visit. Black dashed line represents cut off value of 1.08µmol/l.


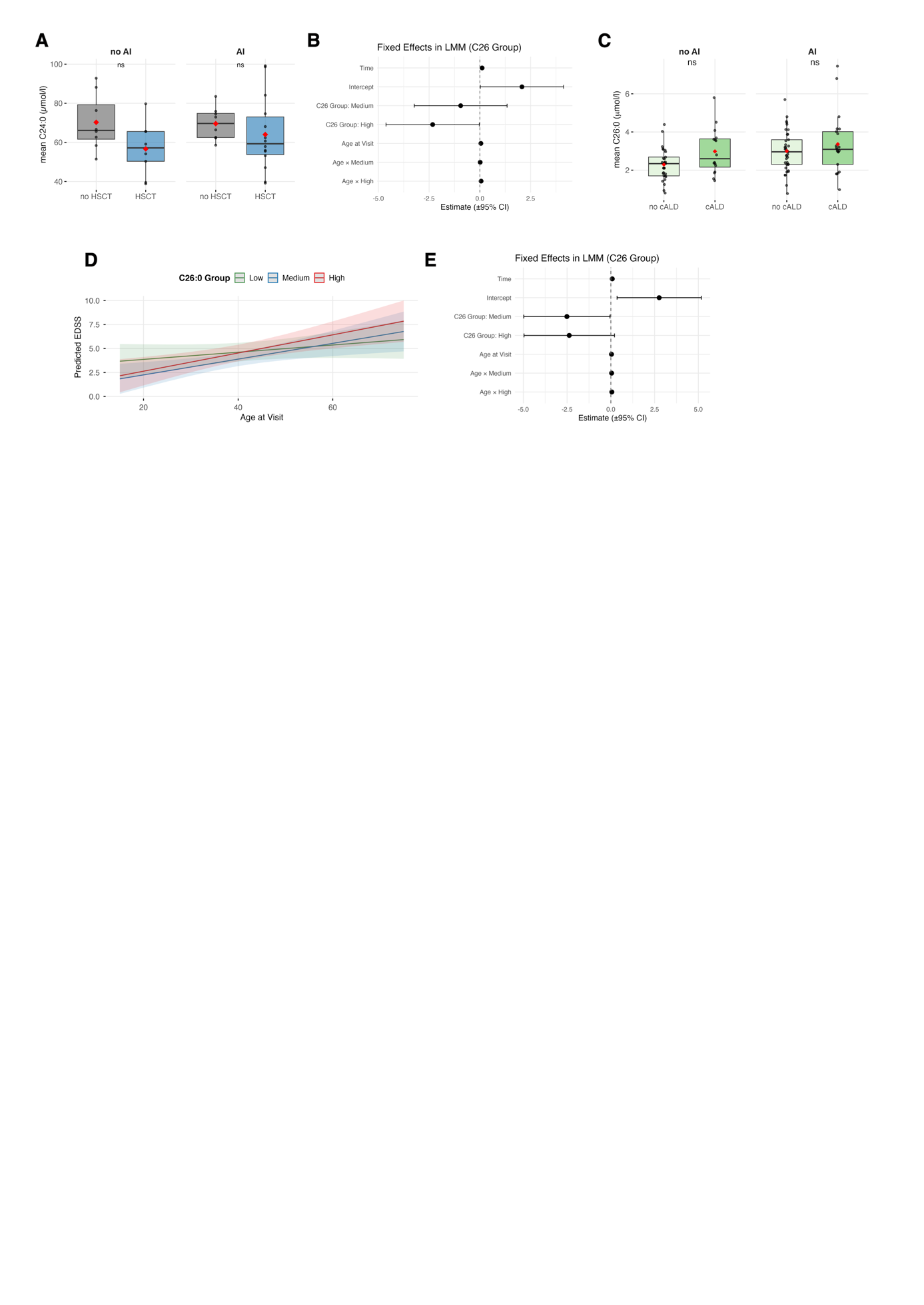


**Supplementary Figure 3:** A: Mean C24:0 in µmol/l in arrested cALD patients without nutritional treatment receiving HSCT or spontaneously arrested based on occurrence of AI or not. No AI/noHSCT: 70.26±14.39, no AI/HSCT 56.67±13.03, p= 0.058; AI/no HSCT: 69.57±8.47, AI/HSCT 64.02±19.16, p=0.45. B: Fixed effects of Linear mixed model analyzing EDSS progression by age based on low (<1.5µmol/l), medium (1.5-2.5µmol/l) or high mean C26:0 (>2.5µmol/l) in male patients. C: Mean C26:0 in µmol/l split by the presence or absence of AI and cALD (untreated): No AI/no cALD: 2.45 ± 1.01, No AI/cALD: 3.26 ± 1.68, p= 0.09; AI/no cALD: 22.92 ± 0.99, AI/cALD: 3.13 ± 1.03, *p*= 0.41. D: Predicted EDSS based on age at visit in patients treated with Lorenzo’s Oil with low mean C26:0 (< 1.5 µmol/l ); medium mean C26:0 (1.5-2.5µmol/l) and high mean C26:0 (> 2.5µmol/l; p (interaction effect of age at visit and high mean C26:0) = 0.041). E: Confidence Intervals of fixed effects of the linear mixed model analysisng the effects of mean patients’ plasma VLCFA and age on EDSS progression in male patients treated with Lorenzo’s Oil.
